# An audit of adherence to cervical cancer screening guidelines in a tertiary-level HIV clinic

**DOI:** 10.4102/sajhivmed.v24i1.1490

**Published:** 2023-05-26

**Authors:** Jeffrey Bolon, Amy Samson, Natalie Irwin, Lyle Murray, Langanani Mbodi, Sarah Stacey, Nicholas Aikman, Louell Moonsamy, Jarrod Zamparini

**Affiliations:** 1 Department of Internal Medicine, Charlotte Maxeke Johannesburg Academic Hospital, Johannesburg, South Africa; 2 Division of Medical Oncology, Department of Internal Medicine, Faculty of Health Sciences, University of the Witwatersrand, Johannesburg, South Africa; 3 Division of Medical Oncology, Department of Internal Medicine, Charlotte Maxeke Johannesburg Academic Hospital, Johannesburg, South Africa; 4 Division of Infectious Diseases, Department of Internal Medicine, Faculty of Health Sciences, University of the Witwatersrand, Johannesburg, South Africa; 5 Division of Infectious Diseases, Department of Internal Medicine, Charlotte Maxeke Johannesburg Academic Hospital, Johannesburg, South Africa; 6 Division of Gynaecological Oncology, Department of Obstetrics and Gynaecology, Faculty of Health Sciences, University of the Witwatersrand, Johannesburg, South Africa; 7 Division of Gynaecological Oncology, Department of Obstetrics and Gynaecology, Charlotte Maxeke Johannesburg Academic Hospital, Johannesburg, South Africa; 8 Department of Internal Medicine, Faculty of Health Sciences, University of the Witwatersrand, Johannesburg, South Africa; 9 Obstetric Internal Medicine Unit, Department of Internal Medicine, Charlotte Maxeke Johannesburg Academic Hospital, South Africa

**Keywords:** HIV, cervical cancer, prevention, women’s health, oncology

## Abstract

**Background:**

Cervical cancer is the most common malignancy affecting South African women aged 15–44 years, with a higher prevalence among women living with HIV (WLWH). Despite recommendations for a screening target of 70%, the reported rate of cervical cancer screening in South Africa is 19.3%.

**Objectives:**

To investigate the adherence of healthcare workers to cervical cancer screening guidelines in a tertiary-level HIV clinic.

**Method:**

A retrospective cross-sectional record audit of women attending the Charlotte Maxeke Johannesburg Academic Hospital HIV Clinic over a 1-month period.

**Results:**

Out of 403 WLWH who attended the clinic, 180 (44.7%) were screened for cervical cancer in the 3 years prior to the index consultation. Only 115 (51.6%) of those women with no record of prior screening were subsequently referred for screening. Women who had undergone screening in the previous 3 years were significantly older (47 years vs 44 years, *P* = 0.046) and had a longer time since diagnosis of their HIV (12 years vs 10 years, *P* = 0.001) compared to women who had not undergone screening. There was no significant difference in CD4 count or viral suppression between women who had and had not undergone screening.

**Conclusion:**

The rate of cervical cancer screening in our institution is below that recommended by the World Health Organization and the South African National Department of Health.

**What this study adds:** This study highlights the low rate of cervical cancer screening among women living with HIV.

## Introduction

Despite being a preventable disease, cervical cancer is the second most common malignancy affecting South African women, second only to breast cancer, and is the most common malignancy in women aged between 15 and 45 years. Approximately 11 000 women are diagnosed with and 5900 women die from cervical cancer annually in South Africa.^1,2^

Southern Africa has the highest incidence of HIV worldwide, with the overall prevalence in South Africa being 13.9%. However, this percentage is considerably higher in South Africa’s female population with current statistics showing that 24.1% of women of childbearing age are living with HIV.^3^

Women living with HIV (WLWH) are six times more likely to develop cervical cancer compared to HIV-negative women, and the malignancy is classified as an AIDS-defining illness.^4^ Furthermore, WLWH are more likely to progress to more advanced stages of cervical cancer at a younger age compared to women who are HIV-negative. In a cohort of patients in the early stages of the HIV pandemic, it was shown that WLWH presented with invasive cervical cancer up to 10 years earlier than women without HIV infection.^5^

Data from the Charlotte Maxeke Johannesburg Academic Hospital (CMJAH) postnatal clinic showed that 47% of WLWH who had a Papanicolaou (Pap) smear done in the postnatal period had an abnormal smear.^6^ Although the increased risk of cervical cancer seen in WLWH is most likely multifactorial, severe immunosuppression and higher rates of coinfection with high-risk strains of human papillomavirus (HPV) are key factors.^4,7^ Therefore, important strategies in preventing cervical malignancies in WLWH include regular cervical cancer screening followed by appropriate management of abnormal results, HPV vaccination and HIV viral suppression using antiretroviral treatment (ART) with resultant immune reconstitution.^8^

The World Health Organization (WHO) has estimated that to prevent 62 million deaths from cervical cancer over the next 100 years, and effectively eliminate the disease, a cervical cancer screening target of 70% needs to be met by 2030 (in addition to HPV vaccination and treatment targets of 90%).^9,10^ South Africa has set itself the same target of 70% screening coverage; however, this target was not met for the period 2000–2004, nor for the period 2005–2014 according to the National Health Laboratory Service (NHLS) national cytology statistics.^11,12^ Furthermore, it has been shown that coverage of cervical cancer screening in South Africa may be as low as 19.3%.^2^

The South African Society of Obstetricians and Gynaecologists (SASOG) currently recommends that in WLWH cervical cancer screening should start at the time of HIV diagnosis and continue three-yearly in low-resource settings, or annually in high-resource settings, and continue throughout the woman’s lifetime. The South African national guidelines recommend three-yearly screening from the time of HIV diagnosis.^11,12,13^

Considering the association between HIV and cervical cancer we aimed to investigate the adherence of healthcare workers to nationally recommended cervical cancer screening guidelines in a tertiary-level HIV clinic.

## Methods

### Study design

We conducted a retrospective cross-sectional record audit of all female adult patients attending the CMJAH HIV Clinic from 01 October 2020 to 31 October 2020. The CMJAH HIV Clinic is a large tertiary-level HIV clinic that acts as a referral centre for people living with HIV from urban Johannesburg and surrounds. Approximately 10 000 patients are seen per year, 60% of whom are women. A minimum of 214 files needed to be audited to achieve a 95% confidence level with a 5% of margin error. Male patients were excluded from the study as were female patients under the age of 18, and those who had undergone a total hysterectomy.

### Data collection

Data, including demographic data, date of last cervical screening, result of previous cervical screening, record of referral for cervical screening, time since HIV diagnosis, most recent CD4 count, HIV viral load (VL) within the previous year and record of any previously abnormal cervical screening or previous hysterectomy, were captured directly from patient files using Google Forms (Google LLC, Mountain View, California, United States) and subsequently exported into Microsoft Excel 16.67 (Microsoft Corporation, Redmond, Washington, United States) for analysis. Data were not retrieved from the NHLS as the study was an audit of the clinic record. No patient identifying data were collected; however, files were marked once analysed to avoid duplicate data entry.

### Data analysis

Data were analysed using Microsoft Excel 16.67 (Microsoft Corporation, Redmond, Washington, United States) and Prism 8.4 (GraphPad Software Inc., La Jolla, California, United States). Non-parametric statistical tests were used as data were non-normally distributed using the Shapiro-Wilk Normality test. Categorical variables, such as the number of women who had undergone a cervical smear, are presented as percentages and frequencies, and Pearson’s chi-square test was used to analyse differences in categorical data between groups. Continuous variables such as age, CD4 and HIV VL are presented as medians with interquartile ranges (IQRs), and the Mann-Whitney test was used to compare continuous variables between two groups. A *P*-value < 0.05 was considered statistically significant.

### Ethical considerations

Permission to conduct the study was granted by the Research Committee and Head of Internal Medicine at CMJAH. Ethical clearance was obtained from the University of the Witwatersrand Human Research Ethics Committee (Medical) with clearance certificate M2011107. The study was also registered on the National Health Research Database.

## Results

During the period under audit, 430 WLWH were seen at the clinic. Twenty-six women had undergone a previous total hysterectomy and one was under the age of 18 years, resulting in a final cohort of 403 women. The median age of this cohort was 46 years (IQR: 39–52 years). The median time from diagnosis to the index consultation was 132 months (IQR: 84–176 months). Two hundred and thirty women (57%) had a known month and year of HIV diagnosis and the remainder, 173 (43%), only a known year of diagnosis. In the case of woman without a known month of diagnosis the month of diagnosis was assumed to be January for calculation purposes.

A CD4 count was available for 389 women. The median CD4 count for those with available data was 523 cells/mm^3^ (IQR: 345 cells/mm^3^ – 722 cells/mm^3^) and 36 women (8.9%) had a CD4 count of less than 200 cells/mm^3^ at the index consultation. HIV VL data was available for 402 women (99.8%) and most women (*n* = 343, 85%), were virologically suppressed (VL < 50 copies/mL). Twenty-five women (6%) had an unsuppressed VL (> 1000 copies/mL).

Concerningly, only 180 women (44.7%) were noted to have undergone cervical cancer screening in the 3 years prior to the index consultation and 223 (55.3%) had no record of screening in the same period. Among women who had undergone cervical cancer screening in the preceding 3 years, 96 smears (53.3%) were reported as negative for intraepithelial malignancy (NILM), 16 (8.9%) as low-grade squamous intraepithelial lesion (LSIL), 6 (3.3%) as high-grade intraepithelial squamous lesion (HSIL) and 4 (2.2%) had lesions recorded as ‘other’. Fifty-eight (32.2%) women did not have a smear result recorded in their clinic file despite having undergone screening.

The differences between screened and unscreened women are described in Table 1. Women who had undergone cervical cancer screening in the preceding 3 years were significantly older (47 years vs 44 years, *P* = 0.046) and had a longer time since diagnosis of their HIV (144 months vs 126 months, *P* = 0.0011) compared to women who had not undergone a cervical smear. There was no significant difference in CD4 count (529 cells/mm^3^ vs 521 cells/mm^3^, *P* = 0.247) nor rate of viral suppression (86.1% vs 84.3%, *P* = 0.674) between women who had and had not undergone screening.

**TABLE 1:** Comparison of women who were and were not screened for cervical cancer in the 3 years prior to the index consultation.

Variable	Total cohort (*n* = 403)			Screened (*n* = 180)			Not screened (*n* = 223)			*P*
	*n*	%	IQR	*n*	%	IQR	*n*	%	IQR	
Age (years)	46	-	39–52	47	-	41–52	44	-	38–52	0.046**
Time since HIV diagnosis (months)	132	-	84–176	144	-	98–181	126	-	72–170	0.0011**
CD4 count, cells/mm^3^	523†	-	345–722	529‡	-	358–729	521§	-	306–714	0.247**
HIV viral load < 50 copies/mL	343	85.3	-	155	86.1	-	188	84.3	-	0.674*
HIV viral load > 50 copies/mL	59	14.7	-	25	13.9	-	34	15.4	-	-

Note: *P*-value calculated by the Mann-Whitney test (**) and Fisher’s exact test (*).

IQR, interquartile range.

† , Total cohort out of *n* = 389;

‡ , Screened out of *n* = 174;

§ , not screened out of *n* = 215.

In the 223 women who had no record of previous cervical cancer screening only 115 (51.6%) women were noted to have been subsequently referred for screening, as shown in Table 2.

**TABLE 2:** Comparison of women who had not been screened for cervical cancer in the 3 years prior to the index consultation and who were or were not referred for screening.

Variable	Total (*n* = 223)			Referred (*n* = 115)			Not referred (*n* = 108)			*P*
	*n*	%	IQR	*n*	%	IQR	*n*	%	IQR	
Age, years	44	-	38–52	45	-	39–52	44	-	37–51	0.496**
Time since HIV diagnosis, months	125	-	65–168	132	-	92–170	118	-	48–158	0.021**
CD4 count, cells/mm^3^	521†	-	306–714	530‡	-	344–718	519§	-	264–720	0.900**
Viral load < 50 copies/mL	188	84.6	-	103	89.6	-	85	79.4	-	0.041*
Viral load > 50 copies/mL	34	15.4	-	12	10.4	-	22	20.6	-	-

Note: *P*-value calculated by the Mann-Whitney test (**) and Fisher’s exact test (*).

IQR, interquartile range.

† , Total out of *n* = 215;

‡ , referred out of *n* = 110;

§ , not referred out of *n* = 105.

There was no significant difference in the age of women who had and had not been referred for a cervical smear (45 years vs 44 years, *P* = 0.496); however, women who were referred for a cervical smear had a median time since HIV diagnosis that was longer than those who were not referred for a smear (132 months vs 118 months, *P* = 0.021). There was no significant difference in CD4 count (530 cells/mm^3^ vs 519 cells/mm^3^, *P* = 0.900) between the two groups; however, there was a significantly higher rate of viral suppression in women who were referred compared to those who were not (89.6% vs 79.4%, *P* = 0.041).

## Discussion

Women living with HIV have a significantly increased risk of cervical cancer. Ongoing population-wide efforts to detect non-invasive disease are recommended across national and international guidelines.^10,11^ In recent years, public health measures to improve accessibility to cervical cancer smear testing for all South African women, and in particular those living with HIV, have been implemented. However, data evaluating adherence and uptake of guideline-recommended screening are lacking.^12^

In this audit, we report on 403 WLWH attending a large HIV clinic in a tertiary centre with direct access to Gynaecology services, in Johannesburg. We show that the target of the National Programme for Cervical Cancer Screening was not met. Fewer than half (44.7%) of the women seen during the study period had undergone screening for cervical cancer within the preceding 3 years. In a similar cross-sectional study among WLWH in Uganda, 44% of women had ever been screened for cervical cancer, with 16.1% having been screened in the preceding year.^14^ While the screening rate in our clinic is well above the national cervical screening rates previously published, it fails to meet both WHO and national guidelines for cervical cancer screening and raises concerns for the cervical cancer screening programme in smaller clinics at both primary care and district level.

Women who had undergone screening for cervical cancer were significantly older than those who had not undergone screening. This may reflect adherence to national guidelines for women without HIV (i.e. screening at age 30, 40 and 50 years) and lack of knowledge of the HIV-specific guidelines. While this result is statistically significant it may not be clinically relevant; considering the median age in both groups was greater than 40 years.

We observed that women who had undergone screening were more likely to have been living with HIV for longer, suggesting that women in care for longer are more likely to be aware of the need for cervical cancer screening and may request referral or self-refer for screening. This is similar to a finding in an Ethiopian study where uptake of cervical cancer screening was significantly higher in women who had been living with HIV for 10 years or longer.^15^ Individuals who are in HIV care for longer also have more opportunity to be referred for cervical cancer screening. Linked to this is the correlation between viral suppression and referral for cervical screening: clinicians may assess women who are virally suppressed as needing less HIV-related care, providing more time to discuss health promotional measures. Among those with no record of previous cervical cancer screening, just over half were subsequently referred for screening. Those who were referred for screening were more likely to be virologically supressed and had been living with HIV for longer, again suggesting that repeat and long-term contact with the healthcare system increases the likelihood of implementation of health promotional measures.

It was not possible to assess reasons for lack of referral in this study as a formal assessment through staff interviews has not taken place. However, women requiring cervical screening are referred to their local (primary care) clinic and there is no standard form or referral letter in use for this. This may reflect an extra administrative burden where clinicians are required to write a referral letter for their patients to have cervical screening. Sigfrid and colleagues suggest that integrating HIV care and cervical cancer screening is both ‘feasible as well as acceptable to women living with HIV’.^7^ Considering this, a further reflection is the lack of a ‘single-visit’ approach for both HIV care and cervical cancer screening. Women are required to attend a primary health clinic for their cervical screening, as noted above, placing an additional time and financial burden on them. Further studies are needed to evaluate if the establishment of a dedicated cervical screening service within our hospital for women at high risk would improve screening rates as it has in other studies.^16,17,18^ Considering the prevalence of HIV in South Africa and the known links between HIV and cervical cancer, an HIV clinic serves as an ideal site for cervical cancer screening. Alternatively, an organised referral system to a dedicated screening facility would greatly improve the number of women referred for screening – this could be initiated through utilisation of currently available gynaecology services in our hospital.

Finally, we are encouraged by the number of WLWH in our clinic who had a recent VL and by the rate of viral suppression among WLWH in our clinic.

### Limitations

Our study was limited by its retrospective nature in that incomplete patient notes may have limited the amount of information captured from each file – women may have been referred for or undergone cervical cancer screening but not had this noted in their file. Additionally, the NHLS system was not checked when results were not recorded in the patient record, thus several patients may have been screened with the result not recorded in the patient record.

## Conclusion

Our study suggests poor adherence to guideline-recommended cervical cancer screening among WLWH in a single specialised centre. The care of people living with HIV is a complex and multifaceted task involving treatment of existing pathology and screening for co-morbid conditions, including cervical cancer. Although this study exhibited promising rates of viral suppression, that is not the only objective of HIV care. Our audit has highlighted substantial gaps in cervical cancer screening as part of the overall management of WLWH at a tertiary-level. This raises concerns for the cervical cancer screening programmes at other tertiary hospitals and, perhaps, more so at lower-level facilities. Furthermore, it highlights the challenges associated with a compartmentalised approach to HIV care with different tasks allocated to different facilities. This ultimately adds further time and financial burdens onto the patient and increases the risk of poor adherence. The reasons for these gaps in care are unclear; however, this audit may serve as a baseline reference to necessitate an intervention and prompt future investigation and quality improvement audits to improve overall care and patient outcomes.
